# Proliferating effect of radiolytically depolymerized carrageenan on physiological attributes, plant water relation parameters, essential oil production and active constituents of *Cymbopogon flexuosus* Steud. under drought stress

**DOI:** 10.1371/journal.pone.0180129

**Published:** 2017-07-14

**Authors:** Minu Singh, M. Masroor A. Khan, Moin Uddin, M. Naeem, M. Irfan Qureshi

**Affiliations:** 1 Plant Physiology Section, Department of Botany, Aligarh Muslim University, Aligarh, India; 2 Proteomics and Bioinformatics Lab, Department of Biotechnology, Jamia Millia Islamia, New Delhi, India; Huazhong Agriculture University, CHINA

## Abstract

Carrageenan has been proved as potent growth promoting substance in its depolymerized form. However, relatively little is known about its role in counteracting the adverse effects of drought stress on plants. In a pot experiment, lemongrass (*Cymbopogon flexuosus* Steud.), grown under different water stress regimes [(100% field capacity (FC), 80% FC and 60% FC)], was sprayed with 40, 80 and 120 mg L^-1^ of gamma irradiated carrageenan (ICA). Foliar application of ICA mitigated the harmful effects of drought stress to various extents and improved the biochemical characteristics, quality attributes and active constituents (citral and geraniol) of lemongrass significantly. Among the applied treatments, ICA-80 mg L^-1^ proved the best in alleviating detrimental effects of drought. However, drought stress (80 and 60% FC), irrespective of the growth stages, had an adverse impact on most of the studied attributes. Generally, 60% FC proved more deleterious than 80% FC. At 80% FC, application of ICA-80 mg L^-1^ elevated the essential oil (EO) content by 18.9 and 25%, citral content by 7.33 and 8.19% and geraniol content by 9.2 and 8.9% at 90 and 120 days after planting (DAP), respectively, as compared to the deionized-water (DW) spray treatment (80% FC+ DW). Whereas, at 60% FC, foliar application of 80 mg L^-1^ ICA significantly augmented the EO content by 15.4 and 17.8% and active constituents *viz*. citral and geraniol, by 5.01 and 5.62% and by 6.06 and 5.61% at 90 and 120 DAP, respectively, as compared to the control (water-spray treatment).

## Introduction

In recent past, use of radiation-processed polysaccharides (RPPs) has attracted the agricultural scientists. Gamma irradiation of natural polysaccharides results in oligomers with low molecular weight, which have proved as potent growth-promoters of plants. These RPPs have the capability of several defence responses in plants [1, 2]. Their effectiveness depends on their molecular size. Application of RPPs, viz. irradiated forms of carrageenan, alginate, chitin, and chitosan, have been reported to induce various kinds of bioactivities such as augmentation of plant growth and productivity, amelioration of anti-microbiological activities in plants and suppression of heavy metal stress [3–11]. It is a novel and emerging technology in the field of agricultural sciences to exploit complete genetic potential of crop plants in terms of growth, yield, and quality.

Carrageenans, the algal polysaccharides, are the sulphated galactans consisting of 1,3 and 1,4 linked galactose residues, derived from various species of red algae (Rhodophyceae). On the basis of number and position of sulphate groups, carrageenans are mainly classified as kappa (k-), iota (i-) and lambda (λ-). Gamma irradiated carrageenan (ICA) has concrete application in agriculture, used either hydroponically or through foliar sprays as plant growth promoter. As per reports, foliar application of ICA enhanced the length of root in potato [12], shoot and root length in rice [13], and increased number of leaves, shoot length and fresh weight of pechay (*Brassicarapa*) [9]. It exerts spectacular effects on physiological activities leading to enhanced productivity of plants [14–17, 10, 11]. Recently, it was reported by Maningas et al., [18] that ICA increased the rice yield up to 60%. In fact, low molecular weight carrageenan (ICA) has also been demonstrated as an effective plant protector against environmental stresses [19]. In context with the present study, Saraet al., [20] reported the increase in osmotic adjustment and soluble proteins in castor bean (*Ricinus communis* L.) under drought stress in response to foliar application of ICA. Keeping these reports in mind, we hypothesized that ICA might also ameliorate the performance of lemongrass under water-stress conditions. However, relatively meager information is known about the role of RPPs in modulating the adverse effects of drought stress on plants. Drought is among the most significant manifestations of abiotic stress in plants [21]. It is well-known to alter a plenitude of physiological and biochemical processes, thus limiting the plant growth and biomass production. Water deficit causes declination in photosynthetic rate, which could most likely be attributed to the reduced activity of RuBisCo, the reduction in stomatal conductance and decline in availability of CO_2_to the site of photosynthesis [22–24]. Drought stress at the cellular level disturbs the water balance of plant, thereby resulting in decreased water potential of cells. Different metabolites, which help in lowering of osmotic potential get accumulated during stress [25]. Loss in the crop yield caused by drought stress perhaps exceeds the losses from all other causes, as both the severity and duration of the stress are critical for crop performance under stress.

Aromatic grasses are an important group of crops, having hardy nature; these plants are considered appropriate for cultivation in barren lands and also for reclamation of these lands through their established soil binding properties [26]. *Cymbopogon* is an important genus of aromatic grasses, species of which are cultivated for their essential oils; lemongrass (*Cymbopogon flexuosus*) is one of them. It is a highly stress tolerant plant that has wide adaptability and carries inherent ability to withstand adverse environmental conditions as compared to traditional agriculture crops [27, 28]. It is an extensive source of essential monoterpene oil(s) and secondary metabolites, particularly citral and geraniol, which constitute the key constituents of numerous chemical, pharmaceutical and perfumery industries and, thus, contributes significantly in agrochemical trade globally.

Considering the aforesaid facts, the current study was planned with an aim to investigate whether the foliar application of ICA could partially or fully mitigate the detrimental effects of drought and ameliorate the plant growth, essential oil (EO) concentration, contents of EO active constituents, productivity and quality of lemongrass under soil water-deficit conditions.

## Materials and methods

### Plant materials and growth conditions

A net-house experiment with simple randomized design was performed on lemongrass (Variety: ‘Krishna’) at the Botany Department, A.M.U., Aligarh, U.P. (India). Lemongrass slips were obtained from the Central Institute of Medicinal & Aromatic Plants (CIMAP), Lucknow. The field capacity (FC) of the experimental soil (100%, 80% and 60%) was maintained in order to ascertain the water deficit at different water stress levels. Before transplanting, a standardized dose of N, P and K was also given to the soil. Physico-chemical characteristics of the soil were: texture- sandy loam, pH 8.12, E.C. 0.37 dS/m, organic carbon 1.09%, and available (mineralizable and water soluble) N, P and K 159, 36.9 and 49.5 mg kg^-1^ of soil, respectively. The samples of soil were analyzed at the Soil-Testing Laboratory, Indian Agricultural Research Institute, New Delhi (India).

### Procedures for gamma irradiation (GI) and gel permeation chromatography (GPC)

**Fig 1:**
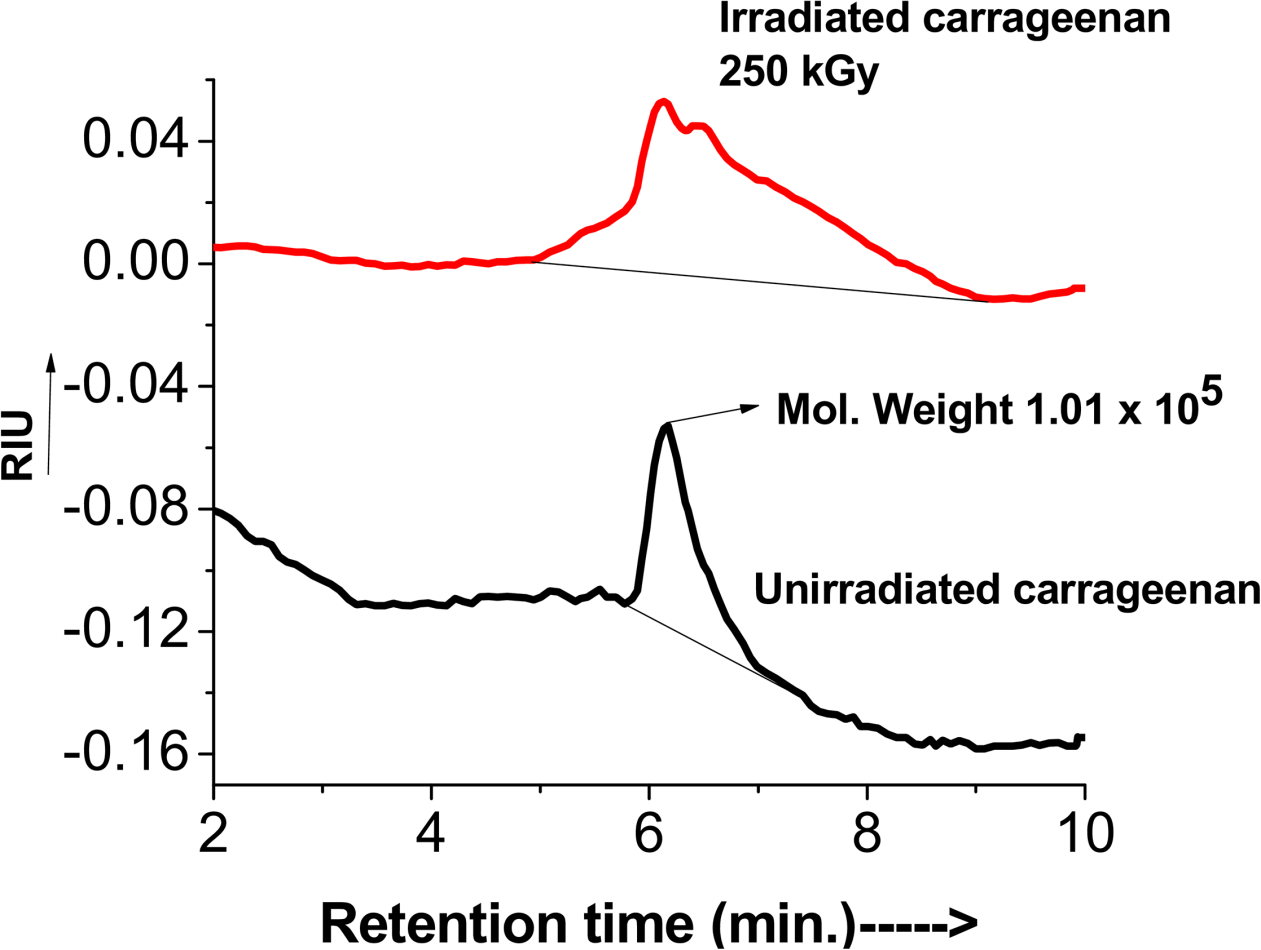
Molecular weight distributions of un-irradiated and irradiated carrageenan. The ICA profile has fraction eluting at higher retention time in comparison to un-irradiated sample, indicating formation of lower molecular weight fragments in the 250 kGy irradiated sample. This fraction also contained less than 20,000 molecular weight oligomers of ICA, which might be responsible for plant growth promotion in this study. The molecular weight of the un-irradiated commercial k-carrageenan was estimated to be about 100,000.

Solid material of k-carrageenan was purchased from Sigma Aldrich, USA. Carrageenan samples were irradiated in Co-60 Gamma Chamber, GC-5000 at BARC, Mumbai, India, at the dose rate of 2.4 kGy/h; the carrageenan samples were irradiated to the total dose of 250 kGy. GPC of carrageenan samples (Fig 1) was done on DIONEX ULTIMATE 3000 machine and the experimental conditions were as follows: mobile phase-water, flow rate-1.5 mL/min, column PL-Aquagel, mixed bed column, 300 mm×10 mm, 20 μL loop injection. The molecular weight of the un-irradiated commercial k-carrageenan sample was estimated to be about 100,000. Polyvinyl alcohol polymers of known molecular weight were used as standards. Radiation dose of 250 kGy was chosen, as no significant change in molecular weight was reported beyond this dose in solid state irradiation of k-carrageenan [12]. Foliar treatments of different concentration of ICA were prepared using double distilled water (DDW).

### Experimental design

The pot experiment was conducted according to the simple randomized design. Drought stress treatments were given to plants when they were fully established (30 DAP) in the pots. There were three moisture regimes, viz. 100% field capacity (100% FC: control), 80% FC (80% FC: mild stress) and 60% FC (60% FC: moderate stress). Each treatment was replicated four times for both the sampling stages [90 and 120 days after planting (DAP)]. To determine a particular parameter with respect to a particular treatment, leaf sample was collected from four different pots (Replicates). Each pot (having a single plant) was considered as a replicate. After the analysis of the desired physiological parameter, the mean value was calculated. Rain-out shelter was used to protect the plants from rain-water and maintain the water stress treatments mentioned above during rainfall period. Weeds were eradicated as and when required. Rotation of experimental pots and treatments was exercised every week to neutralize any positional-effect of pots within the treatments. After theimposition of drought stress, foliar spray of different doses of gamma-irradiated and un-irradiated carrageenan were applied regularly at 15 days interval. Plants were sampled for various physiological, biochemical, quality and yield attributes at 90 and 120 DAP so as to evaluate the consistency of results at the two stages (Table 1).

**Table 1:** The summary of experimental treatments given to *Cymbopogon flexuosus*.

TREATMENTS	DETAILS
T_1_	100% FC + deionized water (DW)
T_2_	100% FC + un-irradiated carrageenan (UICA)
T_3_	100% FC + ICA-40 (40 mg L^-1^ irradiated carrageenan) (ICA)
T_4_	100% FC + ICA-80 (80 mg L^-1^ ICA)
T_5_	100% FC + ICA-120 (120 mg L^-1^ ICA)
T_6_	80% FC + deionized water
T_7_	80% FC + un-irradiated carrageenan
T_8_	80% FC + ICA-40 (40 mg L^-1^ ICA)
T_9_	80% FC + ICA-80 (80 mg L^-1^ ICA)
T_10_	80% FC + ICA-120 (120 mg L^-1^ ICA)
T_11_	60% FC + deionized water
T_12_	60% FC + Un-irradiated carrageenan
T_13_	60% FC + ICA-40 (40 mg L^-1^ ICA)
T_14_	60% FC + ICA-80 (80 mg L^-1^ ICA)
T_15_	60% FC + ICA-120 (120 mg L^-1^ ICA)

### Determination of physiological and biochemical characteristics

#### Total leaf chlorophyll content

In fresh leaves the total chlorophyll content was determined by the method of Lichtenthaler and Buschmann [29]. Leaf-content of the photosynthetic pigment (chlorophyll) was expressed as mg g^-1^ FW.

#### Activities of carbonic anhydrase (CA) and nitrate reductase (NR)

Activities of CA and NR were estimated in this study because the activity of carbonic anhydrase and nitrate reductase is the indicator of rate of photosynthesis and the efficiency of nitrogen metabolism, respectively. The activities of these enzymes may be directly or indirectly related to plant growth and essential oil production of lemongrass.

#### Carbonic anhydrase activity

Carbonic anhydrase activity was measured in fresh leaves, using the method as described by Dwivedi and Randhawa [30]. The enzyme was expressed as μmol CO_2_ kg^-1^ leaf FW s^-1^.

#### Nitrate reductase activity

Nitrate reductase activity was determined by the intact tissue assay method developed by Jaworski [31]. It was expressed as nmolNO_2_ g^-1^ Fh^-1^.

#### Leaf water potential (WP)

Water potential was measured in fresh leaf samples using the PSYPRO water potential system, WESCOR, Inc., Logan, UT, USA.

#### Leaf osmotic potential (OP)

The same leaf, used for water potential measurement, was also used for the determination of osmotic potential, employing a vapour pressure osmometer (WESCOR 5500, Inc Logan, UT, USA).

#### Leaf turgor potential (TP)

Leaf TP was calculated as the difference between water potential (WP) and osmotic potential (OP) using the following formula: TP = WP–OP.

#### Osmotic adjustment (OA)

Osmotic adjustment was calculated as the difference in osmotic potential (Ψs), measured at full turgor, between control and stressed plants.

#### Leaf relative water contents (RWC)

The youngest fully developed leaves from each plant were taken, and then fresh weight of the leaves was recorded. All the leaf samples were then immersed in distilled water for 8 h, followed by measuring the leaf turgid weight after surface-drying the excess water using blotting paper. The leaf samples were oven-dried at 70°C for 24 h, and their dry weight was recorded. RWC was calculated using the following equation:

$$RWC\;(\%)=\frac{Leaf\;fresh\;weight-Leaf\;dry\;weight}{Leaf\;turgid\;weight-Leaf\;dry\;weight}x\;100$$

#### Proline content

Proline content was determined in fresh leaf samples by the method of Bates et al., [32]. The results were calculated by comparing the values with a standard curve plotted by using pure proline and were expressed in μmol proline g^-1^ of fresh leaf tissue.

#### Activities of anti-oxidative enzymes (catalase and peroxidase)

For the extraction of the enzymes, leaf tissue (0.5 g) was homogenized with 5 mL of 50 mmol of phosphate buffer (pH 7.0) containing 1% insoluble polyvinylpyrrolidone. The homogenate was centrifuged at 15,000 rpm for 10 min; the supernatant was used as the source of enzyme. The extraction was carried out at 4°C. Peroxidase and catalase activities were assayed following the procedure described by Chance and Maehly [33]. Catalase activity was estimated by titrating the reaction mixture, consisting of phosphate buffer (pH 6.8), 0.1 M H_2_O_2_, enzyme extract, and 2% H_2_SO_4_, against 0.1 N potassium permanganate. The reaction mixture for measuring peroxidase activity consisted of pyrogallol phosphate buffer (pH 6.8), 1% H_2_O_2_, and enzyme extract. Change in absorbance, due to catalytic conversion of pyrogallol to purpurogallin, was noted at intervals of 20s for 2 min at 420 nm. A control set was employed using distilled water instead of enzyme extract.

### Determination of quality and yield attributes

#### Compositional analysis of essential oil (EO)

Extraction and quantification of the lemongrass EO was carried out according to Guenther [34]. EO content in the leaves (100 g) was obtained by distillation of the content for 3 h, using Clevenger’s apparatus (Borosil, India). The extracted oil was dried over anhydrous sodium sulfate and preserved in sealed glass vials at 4°C for Gas Chromatography (GC analysis) of the oil.

#### Gas chromatography (GC) analysis

The contents of active constituents (citral and geraniol) present in the EO were determined using gas chromatography (Perkin Elmer Auto system XL SAIF, CDRI, Lucknow, India) equipped with the SE-30 stainless steel column (10 ft packed), flame ionization detector and integrator. Nitrogen was used as the carrier gas. GC temperature schedule was as follows: detector temperature, 250°C; oven temperature, 160°C; injector temperature, 250°C. The sample size was 2 μL invariably. The identification of active constituents (citral and geraniol) was based on retention time; the peaks of active constituents were quantified comparing with the peaks from reference standards reported in the literature and, thereafter the contents (%) of active constituents were calculated [35].

### Yield characteristics

Four plants from each treatment were uprooted carefully and washed with tap water to wipe off all adhering foreign particles. Thereafter, the plants were surface-dried using blotting sheets. Plant herbage yield was determined weighing the total plant biomass excluding the roots. Essential oil-yield per plant was calculated using oil content (percent) and the herbage yield per plant [5].

### Statistical analysis

The data were statistically analyzed by SPSS-17 statistical software (SPSS Inc., Chicago, IL, USA). Fisher’s least significant difference (LSD) was calculated in order to differentiate the means at *p*< 0.05 significance level.

## Results

Till date, there is no account of information about the mitigating effects of foliar spray of ICA on this medicinally important essential oil bearing plant under varied levels of drought stress. Therefore, it could be adjudged as a unique finding of its kind, revealing the effect of ICA on herbage yield, biochemical attributes, leaf water-relation parameters, essential oil production and active constituents of lemongrass in stress conditions.

### Physiological and biochemical parameters

Drought stress (80% FC and 60% FC) considerably reduced the growth and metabolism of lemongrass in terms of physiological and biochemical parameters at both the growth stages, *viz*. 90 and 120 DAP. However, the effect of drought stress was comparatively severe at 60% FC. Most physiological and biochemical parameters were improved by the foliar application of ICA in the presence as well as absence of water stress. Of the applied spray-treatments, ICA-80 (ICA applied at 80 mg L^-1^) proved best in overcoming the adverse effects of drought stress. The effect of un-irradiated carrageenan (40 mg L^-1^) was at par with that of the control (spray of deionized water) for most of these parameters studied, indicating that gamma irradiation stimulated the bioactivity of this natural polysaccharide (carrageenan).

**Fig 2:**
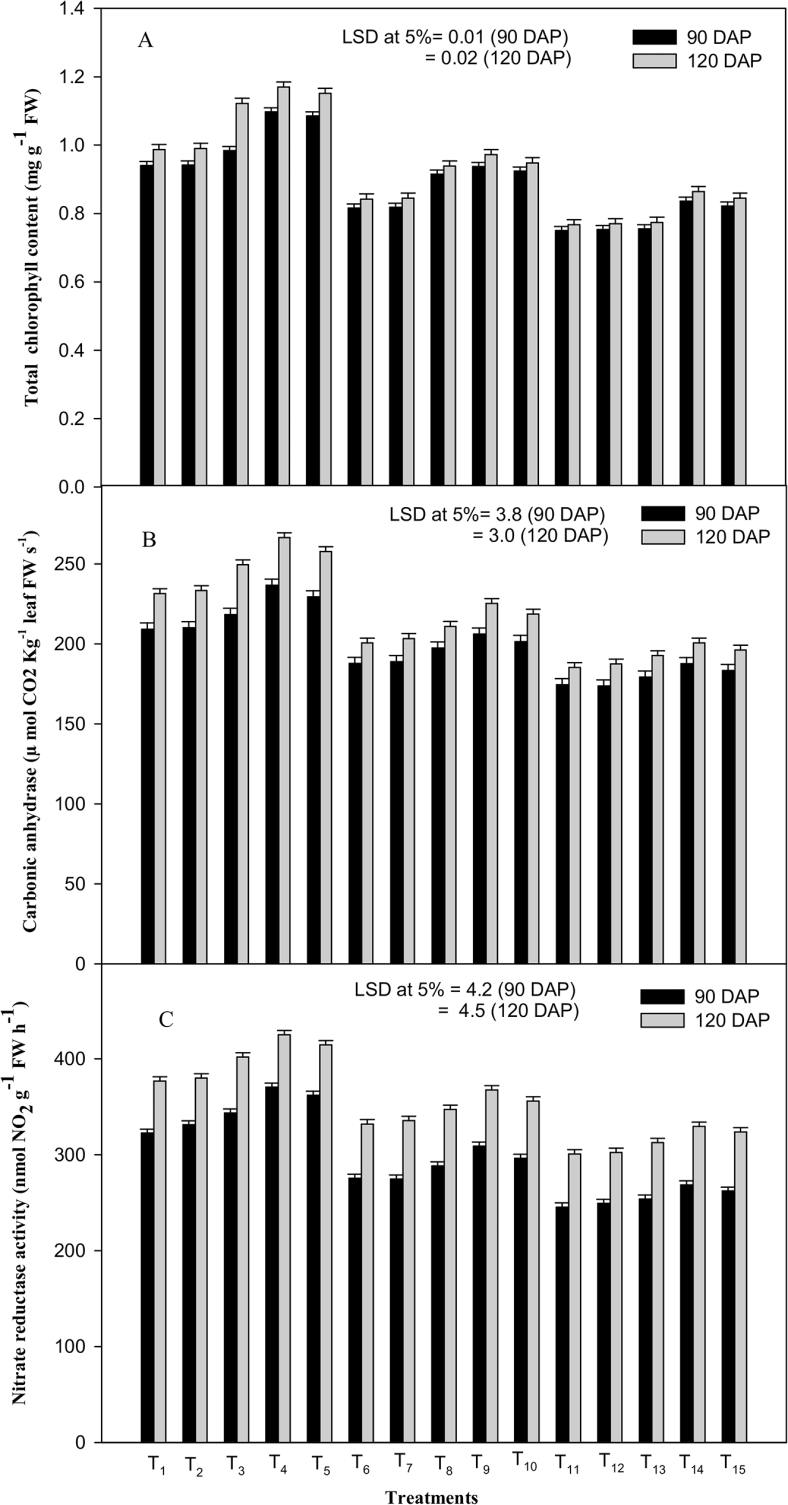
Effects of drought stress and the foliar application of different doses of gamma irradiated carrageenan (ICA) on (A) total chlorophyll content, (B) carbonic anhydrase activity and (C) nitrate reductase activity at 90 and 120 DAP (Days After Planting). Each value represents the mean of four replicates. Bars represent the LSD at 5%.

The total chlorophyll content was significantly decreased by the imposed water stress treatments (80 and 60% FC) irrespective of the growth stage (90 and 120 DAP) (Fig 2A). Exposure of the plant to drought stress at 60% FC was relatively severe, resulting in the decline of leaf chlorophyll content by 20.2 and 22.3%, respectively, at 90 and 120 DAP. While, at 80% FC, it declined the values of leaf chlorophyll content by 13.2 and 14.7%, respectively, at both the growth stages as compared to the control (100% FC). Of the three ICA treatments, ICA-80 proved best in elevating the values of the leaf-chlorophyll content subjected to water stress at 80 as well as at 60% FC. At 80% FC, application of ICA-80 improved the chlorophyll content by 14.8 and 15.4%; while at 60% FC, it improved the chlorophyll content by 11.5 and 12.6% as compared to DW-spray treatment applied at the corresponding drought stress levels (80 and 60% FC) at 90 and 120 DAP, respectively.

Likewise, the activity of CA also showed a significant decline due to drought treatments; however, ICA treatments significantly ameliorated the values of enzyme-activity (Fig 2B). Drought stress at 60% FC was comparatively deleterious; it reduced the activity of CA by 16.6 and 20.0% at 90 and 120 DAP, respectively, as compared to the control (100% FC).While, at 80% FC, drought stress reduced the activity of CA by 10.3 and 13.3% respectively, at both stages of crop growth, as compared to the control (100% FC). Adverse effect of water stress on the activity of CA was ameliorated maximally by ICA-80. At 80% FC, this treatment excelled the activity of CA by 9.8 and 12.3%; whereas, at 60% FC, it enhanced the CA activity by 7.56 and 8.26% as compared to DW-spray treatment (control) applied, at 90 and 120 DAP, respectively.

A similar decreasing trend was also observed in the NR activity, an indicator of nitrogen metabolism by drought treatments. Exposure of the plant to drought stress at 60% FC was more severe, resulting in the decline of NR activity by 23.9 and 20.2% at 90 and 120 DAP respectively, as compared to the control (Fig 2C). However, drought-stress at 80% FC declined the values of NR activity by 14.6 and 11.9%, at 90 and 120 DAP, respectively. Although, subsequent ICA treatments significantly improved the NR activity, the response was more pronounced with ICA-80. It enhanced the NR activity by 12.2 and 10.7% at 80% FC; while at 60% FC, it improved the NR activity by 9.4 and 9.8%, as compared to the control (water-spray treatment applied at the corresponding drought-stress level) at 90 and 120 DAP, respectively (Fig 2C).

**Fig 3:**
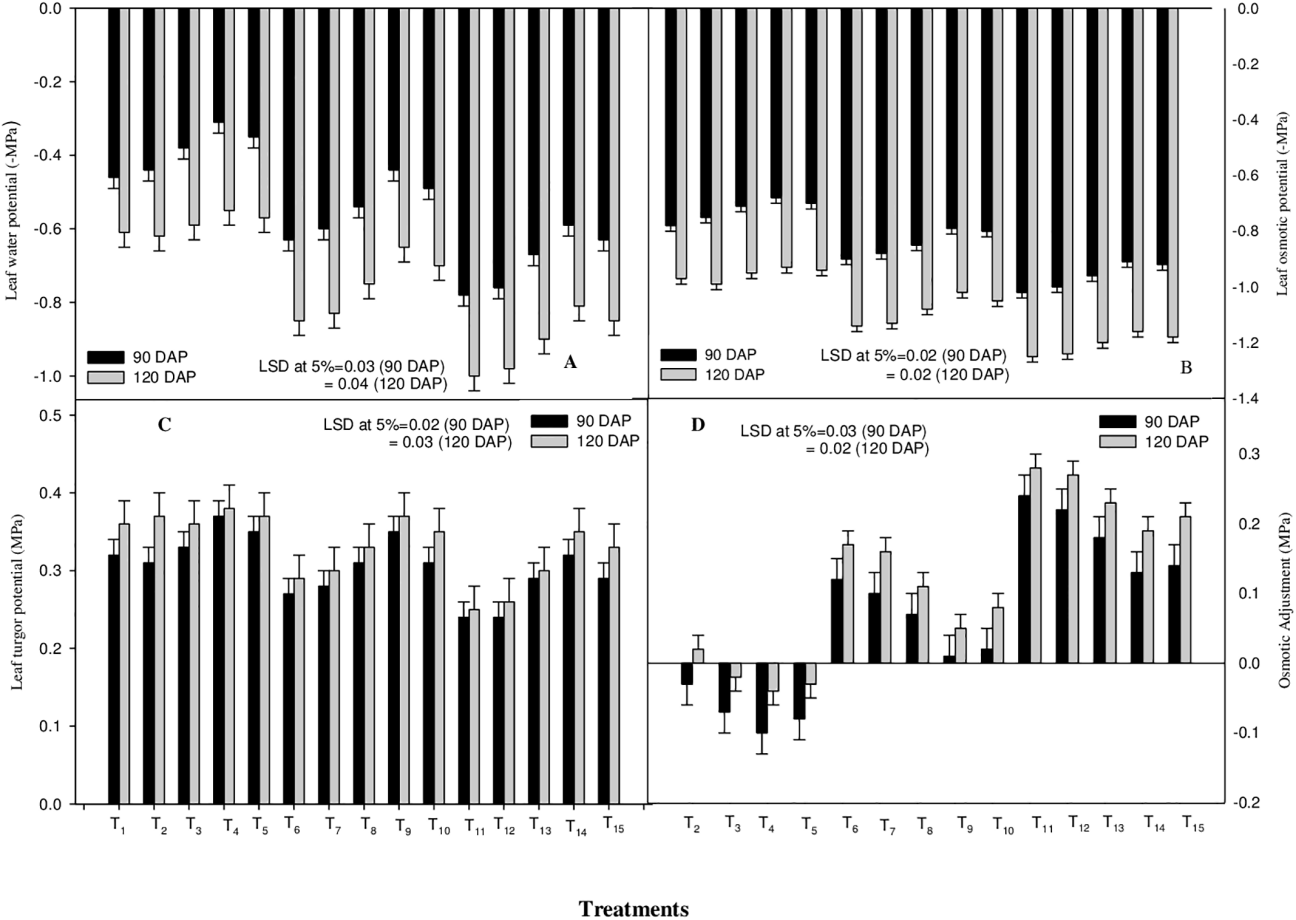
Effects of drought stress and the foliar application of different doses of gamma irradiated carrageenan (ICA) on (A) leaf water potential, (B) leaf osmotic potential (C) leaf turgor potential and (D) osmotic adjustment at 90 and 120 DAP (Days after Planting). Each value represents the mean of four replicates. Bars represent the LSD at 5%.

**Fig 4:**
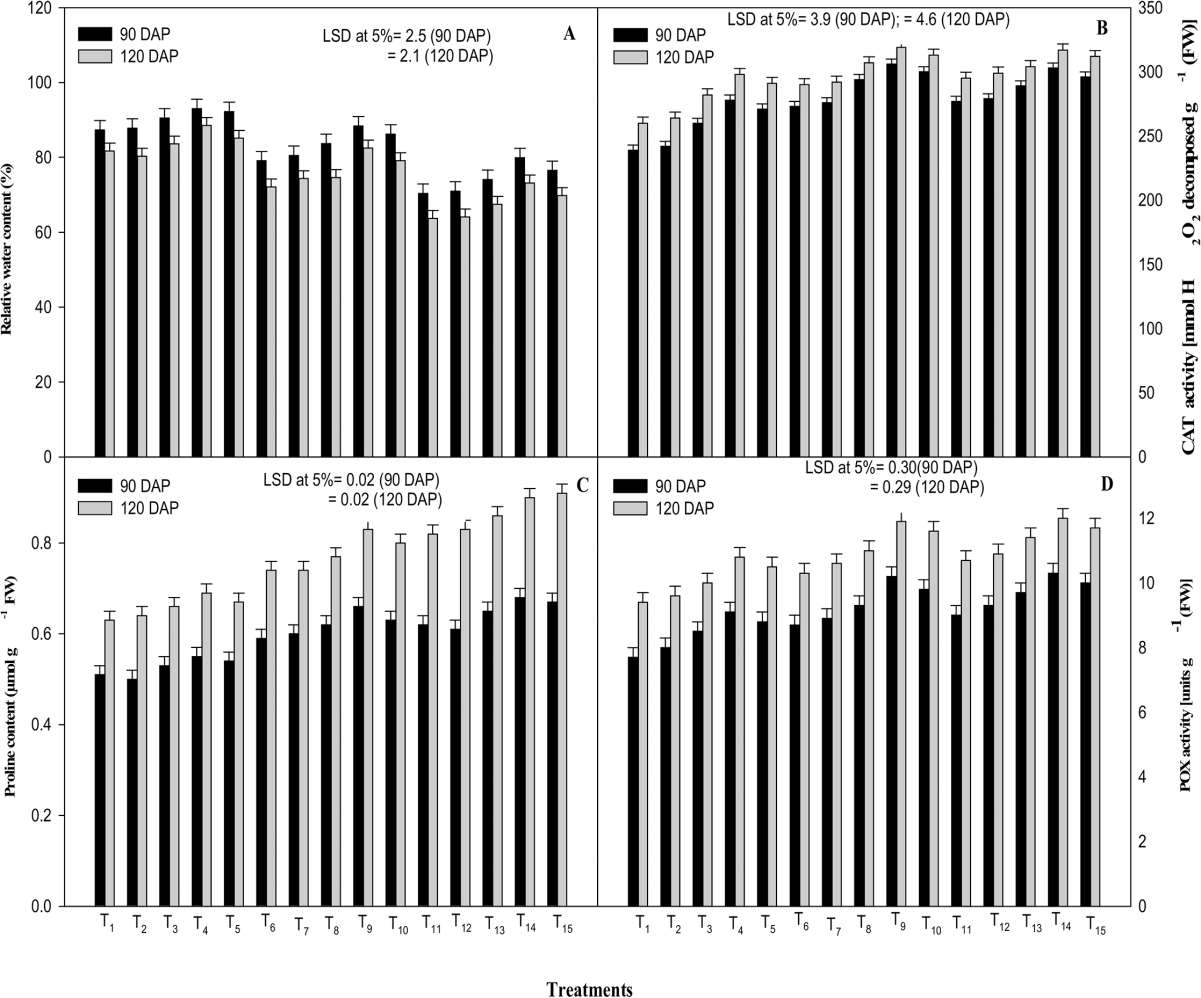
Effects of drought stress and the foliar application of different doses of gamma irradiated carrageenan (ICA) on (A) relative water content, (B) CAT activity, (C) proline content and (D) POX activity at 90 and 120 DAP (Days after Planting). Each value represents the mean of four replicates. Bars represent the LSD at 5%.

Plant water status is an important physiological index for identification of plant response to drought stress. Water relation parameters, viz. leaf water potential (WP), osmotic potential (OP), turgor potential (TP) and relative water content (RWC) were significantly declined by the moisture stress treatments imposed irrespective of the stage of exposure of the plants to water stress (90 and 120 DAP) (Fig 3A, 3B and 3C and Fig 4A). However, osmotic adjustment (OA) showed a reverse trend as, its values were enhanced with increasing level of moisture stress (Fig 3D) at both stages of crop growth. At 60% FC, the water stress was more injurious as compared to that at 80% FC, decreasing the values of WP, OP, TP and RWC by 69.5 and 63.93%, 30.8 and 28.9%, 25.0 and 30.6% and by 19.4 and 22.0% at 90 and 120 DAP, respectively, as compared to the control. At 80% FC, the value with regard to the plant water relation parameters was decreased by 37.0 and 39.3% (WP), 15.4 and 17.5% (OP), 15.6 and 19.4% (TP) and by 9.4 and 11.8% (RWC) at 90 and 120 DAP, respectively, as compared to the control. Moreover, the foliar application of ICA significantly ameliorated the drought induced decrease in respective water relation parameters at 80 as well as 60% FC, the plant response being more pronounced with ICA-80.

Proline content in the leaves was found maximum in moderate stress condition (60% FC), while its lowest amount was recorded in un-stressed condition (Fig 4C). The drought stress at 60% FC increased the values of proline content by 21.6 and 30.2% at 90 and 120 DAP respectively, as compared to the control (100% FC). While, at 80% FC, it ameliorated the proline content by 15.7 and 17.5% respectively, at 90 and 120 DAP as compared to the control (100% FC). Moreover, foliar application of ICA-80 further enhanced the proline content by 11.9 and 12.2% at 80% FC; while at 60% FC, it improved the proline content by 9.7 and 9.9%, as compared to the control (water-spray treatment applied at the corresponding drought-stress level) at 90 and 120 DAP, respectively (Fig 4C).

The activities of catalase and peroxidase enzymes were also augmented under drought stress (80% and 60% FC) both at 90 and 120 DAP, as compared to the control (Fig 4B and 4D). Combined application of ICA and drought stress (80 and 60% FC) had an additive effect on the activity of oxidative stress enzymes (catalase and peroxidase).At 90 DAP, the combined application of ICA-80 and drought stress at 80% FC resulted in 28.0 and 32.5% increase in the activity of catalase and peroxidase, respectively; while, at 120 DAP the combined application of ICA-80 and 80% FC resulted in 22.7 and 26.6% enhancement in the activity of the two enzymes, respectively, over the control (100% FC). At 60% FC, ICA-80 augmented the activity of catalase and peroxidase by 26.7 and 21.9% and by 33.8 and 27.7% at 90 and 120 DAP, respectively, as compared to the control (100% FC).

### Yield and quality attributes

**Fig 5:**
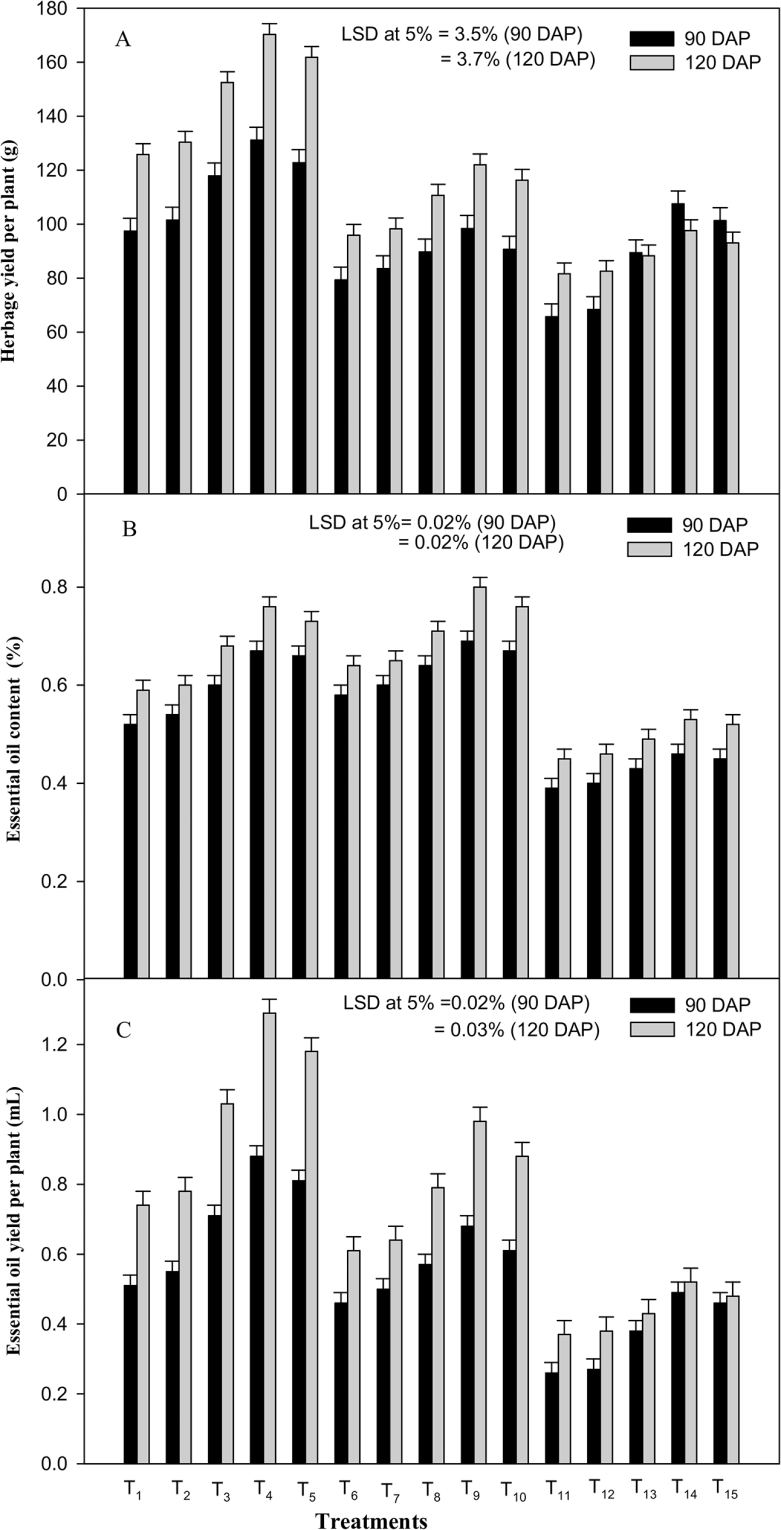
Effects of drought stress and the foliar application of different doses of gamma irradiated carrageenan (ICA) on (A) herbage yield per plant, (B) essential oil content and (C) essential oil yield per plant at 90 and 120 DAP (Days after Planting). Each value represents the mean of four replicates. Bars represent the LSD at 5%.

Drought stress had a detrimental effect on *Cymbopogon* herbage-yield at both the growth stages. Its effect at 60% FC was more deleterious than at 80% FC. Drought stress at 60% FC markedly reduced the herbage yield by 32.5 and 35.1% over the control at 90 and 120 DAP, respectively. While, at 80% FC, it decreased the herbage yield by 18.6 and 23.8% at 90 and 120 DAP, respectively, as compared to the control (100% FC). Moreover, exogenous application of different doses of ICA significantly enhanced the herbage yield under stress and stress free conditions (Fig 5A).

On the other hand, at 80% FC the EO content showed a significant increase of 11.5 and 8.5% at 90 and 120 DAP, respectively as compared to the control (Fig 5B). Whereas, at 60% FC drought stress resulted in the decline of EO content by 25.0 and 23.7% as compared with the control at 90 and 120 DAP, respectively. Although, positive effect of ICA treatments was noted on EO content at both the growth stages. ICA-80 proved best for stressed and stress free plants; it maximally augmented the EO content at 80% FC by 18.9 and 25% and at 60% FC by 15.4 and 17.8% as compared to the control (water-spray treatment applied at the corresponding drought-stress treatments) at 90 and 120 DAP, respectively. The essential oil yield per plant however, showed disparity and was considerably reduced both under mild (80% FC) and moderate (60% FC) water stress during the two growth stages (Fig 5C). Despite this, the deleterious effect of drought stress on the essential oil yield was partially neutralized by the foliar spray of ICA-80. This treatment (ICA-80) enhanced the EO yield per plant by 47.8 and 60.7% at 90 and 120 DAP, respectively, at 80% FC; while, at 60% FC, it augmented EO yield per plant by 30.7 and 32.4%, at 90 and 120 DAP, respectively, as compared to the stressed plants sprayed with DW (Fig 5C).

**Fig 6:**
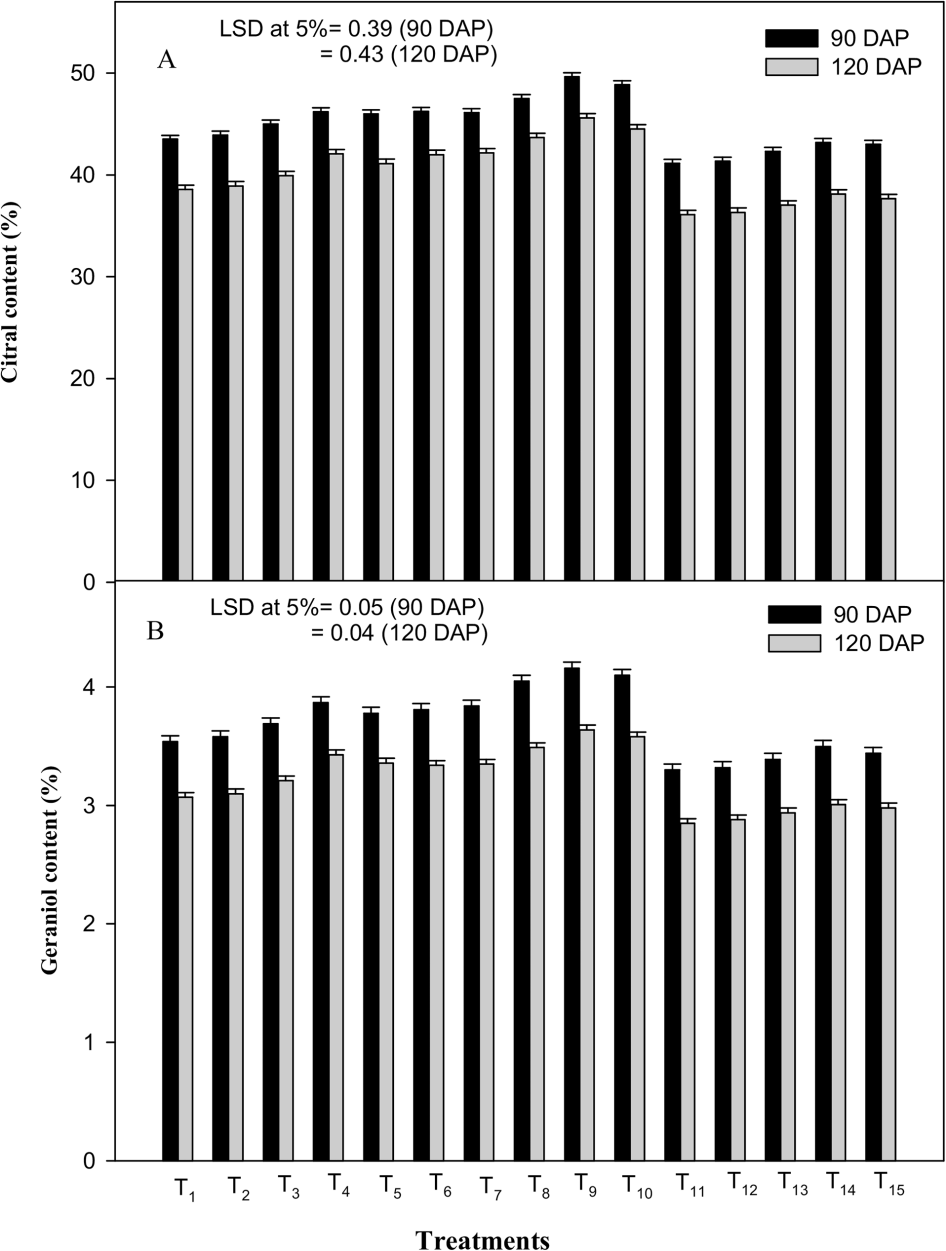
Effects of drought stress and the foliar application of different doses of gamma irradiated carrageenan (ICA) on (A) citral content (%) and (B) geraniol content (%) in essential oil at 90 and 120 DAP (Days after Planting). Each value represents the mean of four replicates. Bars represent the LSD at 5%.

Drought stress at 80% FC increased the citral and geraniol contents by 6.3 and 7.6% at 90 DAP and by 8.9 and 8.8% at 120 DAP, respectively, as compared to the control (100% FC). However, the plants subjected to 60% FC showed a significant decrease of 5.4 and 6.8% at 90 DAP and of 6.4 and 7.2% at 120 DAP in citral and geraniol content, respectively, as compared to the control (100% FC) (Fig 6A and 6B). Moreover, the subsequent ICA treatments (40, 80 and 120 mg L^-1^ of ICA) applied at the two drought stress treatments (80 and 60% FC) significantly improved the level of citral and geraniol contents at both the crop stages. Among the applied spray-treatments, the plant response was comparatively more pronounced with ICA-80. At 80% FC, it enhanced the EO active-constituent by 7.3 and 8.19% (citral) and by 9.2 and 8.9% (geraniol) at 90 and 120 DAP, respectively; while at 60% FC, ICA-80 augmented the citral and geraniol content by 5.01 and 5.62% and by 6.06 and 5.61% at 90 and 120 DAP, respectively, as compared to the deionized-water spray treatment (control) (Fig 6A and 6B).

## Discussion

### Physiological and biochemical parameters

In the present study, it was observed that drought stress (80% and 60% FC) imposed on lemongrass plants, significantly declined the values of leaf chlorophyll content and the activities of CA and NR (Fig 2A, 2B and 2C). It is well established that drought stress induces the accumulation of abscissic acid (ABA) in the leaves [36] that acts as a biochemical adaptation to reduce the water loss during scarcity of water. Accumulation of ABA in the leaves induces the closure of stomata as well as reduces their conductance, thereby affecting the gaseous exchange [37]. Therefore, the plants subjected to drought stress possessed lower intercellular CO_2_ concentration as compared to the control. Chlorophyllase is an enzyme that regulates the turnover of chlorophyll molecules [38]. Water stress is well known to enhance the activity of chlorophyllase, thereby, promoting degradation of the chlorophyll molecules rather than synthesis [39]. It also decreases the biosynthesis of δ-aminolavulinic acid and protochlorophyllide reductase complex [40], involved in chlorophyll biosynthesis. The cumulative suppressive effect of these two reactions, therefore, resulted in the loss of leaf chlorophyll content. In addition,the reduction in leaf chlorophyll content in this investigation could also be ascribed to the decreased activity of chlorophyll synthetase [41]. However, the enhancement in chlorophyll content and mitigation of the negative effect of stress on this parameter could be attributed to the beneficial effect of foliar application of ICA on photosynthesis coupled with that on the entire plant growth. The present results are in conformity with the earlier findings which reported the ICA-mediated increase in photosynthetic parameters *viz*. stomatal conductance, PSII efficiency, chlorophyll *a* and *b* content, and RuBisCo activity [11, 16, 42, 43]. Drought stress also resulted in reduced activities of NR (Fig 2C) and CA (Fig 2B). Decrease in NR activity under water stress might be speculated as a biochemical adaptation to save energy by ceasing nitrate assimilation [44, 45]. Additionally, owing to transpiration and loss of turgor during stress, absorption and transportation of nitrate declines significantly, which may adversely affect the post-translational control factors, including proteolysis and phosphorylation [46, 47], affecting the enzyme activities unfavorably. In the same context, reduction in CO_2_ supply along with its internal concentration due to possible stomatal closure and decreased photosynthetic-rate could result in the reduced activity of CA [48] under water stress. Furthermore, likely inhibition of RuBisCo and PEP carboxylase enzymes under drought stress might also be responsible for decreased activity of CA [49]. As observed by Popova et al., [50] and Singh and Usha [51], the ICA-improved CA activity under the drought stress conditions could be attributed to the improved activity of RuBisCo and PEP carboxylase. The observed positive effect of ICA on the activity of NR under water-stress conditions (Fig 2C) could be ascribed to the role of ICA in lessening the stress-induced damage on plasma membrane permeability. It might have facilitated the enhanced uptake of nutrients including nitrate, improving the NR activity under stress [52]. Beneficial effect of irradiated natural polysaccharides on the activity of these enzymes has earlier been reported [5, 6, 8, 16] on various medicinal and aromatic plants.

Drought stress reduced the capacity of plants to take up water from the soil, which evidently exerted negative effect on plant water relation parameters (WP, OP, TP and RWC) in this investigation. As per results, foliar application of different concentrations of ICA significantly augmented the plant water status parameters under stress and stress-free conditions at both the growth stages (Fig 3A, 3B and 3C and Fig 4A). Water status of plants is mainly determined through osmotic conditions of cells and transportation of water from root to shoot. Osmotic regulation positively influences the level of water potential in assimilating tissues during reduced water transport from root to shoot and, thereby, limits the inhibitory effects of drought stress on photosynthesis [53–55]. The decrease in osmotic potential under water stress could be attributed to the reduced plant-water content that might result in higher concentration of solutes, greater elasticity of tissue, and/or active solutes accumulation [56]. Enhancement in osmotic adjustment (OA) under water stress conditions in the present experiment could be attributed to accumulation of small molecules including organic solutes (soluble sugar, proline, etc.) and inorganic ions (K^+^, Ca^2+^, Mg^2+^, etc.), the cause of greater osmotic adjustment [57]. Generally, during water stress, OA helps in maintaining turgor potential (TP) of both shoots and roots. Improved plant-OA allows the turgor-dependent processes (for instance, the growth and stomatal activity) to carry on gradually at low leaf water potentials [58]. A possible mechanism behind the amelioration in WP, OP, TP and RWC due to foliar application of ICA, under both stress as well as stress-free conditions, might be the ICA-mediated improved membrane stability, which might have reduced the electrolyte leakage; that, in turn, could have improved the plant water relation parameters in this study. As per results, the ICA application might have increased the plant ability to translocate the photosynthates for better osmoregulation (Fig 3A, 3B and 3C and Fig 4A).

Contrary to other parameters, leaf-proline content (Fig 4C) and antioxidant enzymes viz. catalase and peroxidase, (Fig 4B and 4D) showed an escalating response to ICA and/or moisture deficit treatments. The level of antioxidant enzymes and that of the osmolytes, such as proline, are generally improved in plants to maintain osmotic potential and turgor pressure of cell. They also play various roles such as those of membrane stabilizer [59], ROS scavenger [60] and maintenance of proper protein conformation at low leaf water potentials [61]. Moreover, it is also reported that the enzymes responsible for proline biosynthesis gets elevated during water stress, whereas those responsible for its degradation are suppressed [62]. Hence, the improvement in these biochemical parameters could be ascribed to the observed positive effect of drought stress on proline levels in comparison with the control. Additionally, further enhancement in level of proline with the application of ICA could be the result of ICA mediated activation of enzymes involved in proline biosynthesis [43] (Fig 4C).

Increase in the activities of catalase (CAT) and peroxidase (POX) during drought stress may be attributed to the general antioxidative system in plants, which involves regulation of protein synthesis via gene expression [63]. CAT and POX are the principal enzymes that scavenge reactive oxygen species (ROS) in plants, thereby averting the cellular damage under stress conditions [64–66]. Higher activities of antioxidant enzymes during water stress in plants are linked with reduced levels of lipid peroxidation, which is connected to drought tolerance [67]. In this study, the positive effect of ICA on the antioxidant enzymes may perhaps be attributed to its role in transcription and/or translation of the concerned genes [68], facilitating the plant system with synthesis of these enzymes in order to enhance the plant tolerance against drought stress. These results are further confirmed by the findings of Gonzalez et al., [43] which reported the ICA induced increase in antioxidative enzymes *viz*. ascorbate (ASC), glutathione syntheses (GSH) and thiredoxin reductase (TRR)/ thioredoxin (TRX) activities in *Eucalyptus*. Moreover, studies of Tsipali et al., [69] also revealed that phosphorylated and sulfated glucans exhibit higher antioxidant ability, which indicate that polyelectrolytes, such as glucan sulfate (e.g. carrageenan) might have increased scavenging activity. Studies of Siriwardhana et al., [70] further confirms that low molecular weight polysaccharides influence the antioxidant activity.

### Yield and quality parameters

Generally, the decline in yield attributes due to water stress could be ascribed to the reduction in photosynthesis; either by limitation of leaf area expansion, by temporary wilting, by rolling of leaves during severe stress, or by leaf-senescence in early stage [71,72]. Additionally, reduced harvest index (HI) during drought stress is considered to contribute to decreased crop yield. In the present study, occurrence of such factors cannot be ruled out, though the agronomic data in this regard were not recorded, except that of crop herbage yield. Similar results on the effect of water stress on yield and/or biomass reduction of different crops have been reported by other workers regarding different crops [73–75]. The observed positive effect of foliar application of ICA on herbage yield may be traced to its various roles in plant growth stimulation and to the combined effects of numerous underlying stimuli mediated by ICA. Our results strengthen the findings of Naeem et al., [17, 11] and Hashmi et al., [16], who observed an increase in the yield of *Mentha arvensis* and *Foeniculum vulgare* with application of ICA in unstressed conditions.

As per results (Fig 5C), the essential oil (EO) yield per plant showed considerable reduction under mild (80% FC) as well as moderate (60% FC) water stress at both growth stages. Alternatively, the content of EO and that of major EO constituents, viz. citral and geraniol, in lemongrass showed a substantial increase under mild stress (80% FC). Drought stress induced elevation in EO content could be attributed to the increased density of the oil glands in the leaves due to the reduction in leaf area and production of greater amount of metabolites in plants during the stress period. These results corroborate the findings of Fatima et al., [76] and Khalid et al., [77] on different medicinal and aromatic plants under drought stress. Moreover, changes in EO composition (plant’s active constituents) might be ascribed to the stress-mediated effect on the *de novo* synthesis of specific enzymes involved in the biosynthesis of monoterpenes. Similar reports on different plants have been observed by Idrees et al., [49] and Ali et al., [8]. In this investigation, drought-induced reduction in EO yield could be attributed to the overall retardation of plant growth and differentiation under stress condition as noted under several studies [78–80]. Presumably, the deleterious effect of drought stress on the EO yield was partially neutralized by the foliar spray of ICA-80. The observed positive effect of ICA application on EO yield and the contents of its active constituents (citral and geraniol) (Figs 5C, 6A and 6B) might be attributed to ICA-stimulated vegetative growth, populationof leaf oil glands, nutrient accumulation (N and P)and also to the plant metabolismand enzymatic activities responsible for mono or sesqueterpene-biosynthesis, which could have consequently resulted in increased synthesis of metabolites pertaining to EO production under stressed conditions. This conclusion is in accordance with the findings of Sarfaraz et al., [81] on fennel and of Naeem et al., [17, 11] on *Mentha arvensis*. In this study, the ICA-mediated positive effect on the yield of lemongrass EO and its major active constituents, viz. citral and geraniol, under moisture stress, have been reported for the first time.

## Conclusions

Based on the results extracted from this investigation, it could be concluded that gamma-irradiated carrageenan (ICA) proved to be a potent elicitor in improving the metabolism, plant water relation parameters, activities of antioxidant enzymes, contents of EO active-constituents, EO concentration and EO yield (amount per plant) of lemongrass under drought-stress condition (as well as under unstressed condition). Furthermore, this investigation revealed that the cultivation of hardy medicinal plants (like that of lemongrass), in water-deficit areas would be a suitable option in view of the improved proline metabolism, osmoregulation, defense system and the level of active principles. Since, foliar application of ICA paves the way for partial amelioration of drought stress, this novel technique could be recommended for profitable cultivation of lemongrass under unfavorable conditions of moisture stress. However, additional research is required to apprehend the mechanism and mode of action of carrageenan-derived oligomers on plants under water stressed conditions.
